# Antithrombin in Hyperuricemic Nephropathy: Unveiling Its Noncanonical Renoprotective Roles and Therapeutic Repurposing Potential

**DOI:** 10.1159/kbr/adjag005

**Published:** 2026-09-08

**Authors:** Lei Jin, Xiaomin Zhang, Feng Wang

**Affiliations:** Department of Rheumatology, Immunology and Allergy, Shanghai General Hospital, Shanghai Jiao Tong University School of Medicine, Shanghai, China

**Keywords:** Antithrombin, Gout, Nephropathy, Hyperuricemia, Thromboinflammation

## Abstract

**Background:**

The pathogenesis of hyperuricemic nephropathy (HN) is increasingly linked to a self-perpetuating cycle of thromboinflammation. This process begins with hyperuricemia-induced endothelial dysfunction, which activates both the coagulation cascade and inflammatory pathways, notably the pyrin domain-containing protein 3 inflammasome. This creates a vicious cycle of which inflammation promotes coagulation and coagulation products further amplify inflammation, ultimately leading to renal microvascular thrombosis, ischemia, and progressive fibrosis.

**Summary:**

This review critically examines the therapeutic potential of antithrombin (AT) in interrupting this pathogenic axis. AT, a key serine protease inhibitor, is central to physiological anticoagulation. Beyond its canonical role in neutralizing thrombin and factor Xa (FXa), AT exerts potent anti-inflammatory and endothelial-stabilizing effects, primarily mediated through interactions involving its D-helix domain. Compelling preclinical evidence from models of sepsis, ischemia-reperfusion, and diabetic nephropathy demonstrates that AT can attenuate inflammation, improve microcirculation, and reduce fibrosis. We hypothesize that in HN, augmenting AT levels may simultaneously dampen thrombin generation, quell the associated inflammatory response, and protect the endothelium, thereby potentially mitigating the structural drivers of renal decline. These findings suggest that AT might serve as a multi-target candidate for disease modification, pending validation in HN-specific models. Notably, this hypothesis remains untested in HN-specific models, with current evidence extrapolated from related thromboinflammatory conditions. Therefore, this review synthesizes the mechanistic rationale, clearly delineates established science from translational hypothesis, and identifies the crucial experimental gaps – particularly regarding optimal dosing, timing, and direct validation of efficacy in HN – that must be addressed to evaluate the potential of AT repurposing as a candidate targeted therapy for hyperuricemic kidney injury.

## Introduction

Hyperuricemic nephropathy (HN) is a significant form of chronic kidney disease (CKD) caused by abnormalities in purine metabolism. Prior to urate-lowering therapy, up to 25% of gout patients already have renal damage, 20%–40% exhibit reduced renal function, and autopsy findings confirm that renal pathological changes are prevalent in gout patients [1]. Traditional pathophysiological mechanisms have focused on the direct deposition of urate crystals in the renal tubules and interstitium; however, modern perspectives suggest that a systemic “thromboinflammation” network triggered by soluble urate and crystals is key to driving the progression of nephropathy [2]. Epidemiological evidence indicates that elevated uric acid levels increase the risk of thrombotic events and recurrence [3] and that hyperuricemia and gout are independent risk factors for venous thromboembolism, left atrial thrombi associated with atrial fibrillation, and atherosclerotic thrombotic events [4, 5].

Given the well-defined thromboinflammation network in HN, targeting the coagulation system – particularly antithrombin (AT) and its associated anticoagulant and anti-inflammatory mechanisms – has emerged as a promising area for intervention. The anticoagulant system, particularly AT and direct activated coagulation factor X (FXa) inhibitors, has demonstrated organ-protective effects beyond simple anticoagulation in various CKD models [6–9]. Among these, AT is the most important physiological anticoagulant in the body, not only significantly enhancing the inhibitory activity against coagulation factors (particularly FXa and thrombin) by binding to heparin [10], but its D-helix domain also mediates potent anti-inflammatory and immunomodulatory effects, including the induction of prostacyclin release, inhibition of leukocyte activation, and downregulation of pro-inflammatory cytokine expression [11]. However, clinical trials of exogenous AT supplementation in systemic inflammation (such as severe sepsis) have not achieved clear success in the general population [12], suggesting that its efficacy may be highly dependent on specific pathological environments and patient subgroups.

Therefore, a systematic elucidation of the mechanisms of action and therapeutic prospects of the anticoagulation system in HN is of great significance. In this review, we advance the central hypothesis that in HN, augmenting AT levels could simultaneously curb thrombin generation, quell the associated inflammatory response, protect endothelial function, and thereby interrupt the key drivers of progressive renal decline. This hypothesis, rooted in the shared thromboinflammatory pathology, positions AT as a unique multi-target candidate for disease modification. The following sections synthesize the mechanistic rationale, delineate established science from translational hypotheses, and identify crucial experimental gaps.

## Key Pathogenic Mechanisms of Coagulation, Thrombosis, and Inflammation in HN

The progression of HN can be conceptualized as a self-amplifying cascade driven by a “thromboinflammation” circuit [2]. This framework connects the initiating metabolic insult (hyperuricemia) to renal fibrosis [13–15]. Clinically, elevated serum uric acid (≥4.38 mg/dL) is an emerging risk marker for thrombosis, tripling the risk of VTE recurrence [2, 3]. Mechanistically, hyperuricemia initiates endothelial injury and dysfunction, creating a procoagulant, pro-inflammatory vascular phenotype [16, 17]. This altered endothelium serves as a platform that activates both the coagulation cascade and innate immune responses, notably the NOD-, LRR-, and pyrin domain-containing protein 3 (NLRP3) inflammasome [2, 6, 17]. These systems engage in a bidirectional vicious cycle, where inflammation promotes coagulation and coagulation products (e.g., FXa, thrombin) further fuel inflammation via protease-activated receptors (PARs) [6]. This systemic thromboinflammatory state is reinforced by activated cellular components (platelets, erythrocytes, neutrophils) and the complement system. Due to its unique microvascular architecture, the kidney becomes the critical convergence site. The systemic pathology manifests locally as diffuse intrarenal microthrombosis, leading to ischemia and hypoxia. The resulting hypoxic microenvironment, sustained by ongoing inflammatory signals, drives fibrogenic pathways, culminating in glomerulosclerosis and tubulointerstitial fibrosis. Thus, by inducing endothelial dysfunction, oxidative stress, systemic inflammation, and a hypercoagulable state, hyperuricemia forms a “thrombotic-inflammatory” network. The abnormal activation of coagulation serves as the critical link connecting inflammation, microvascular injury, and progressive renal fibrosis in HN (Fig. 1). The following sections detail the evidence and mechanisms supporting each node of this integrative framework.

**Fig. 1.**
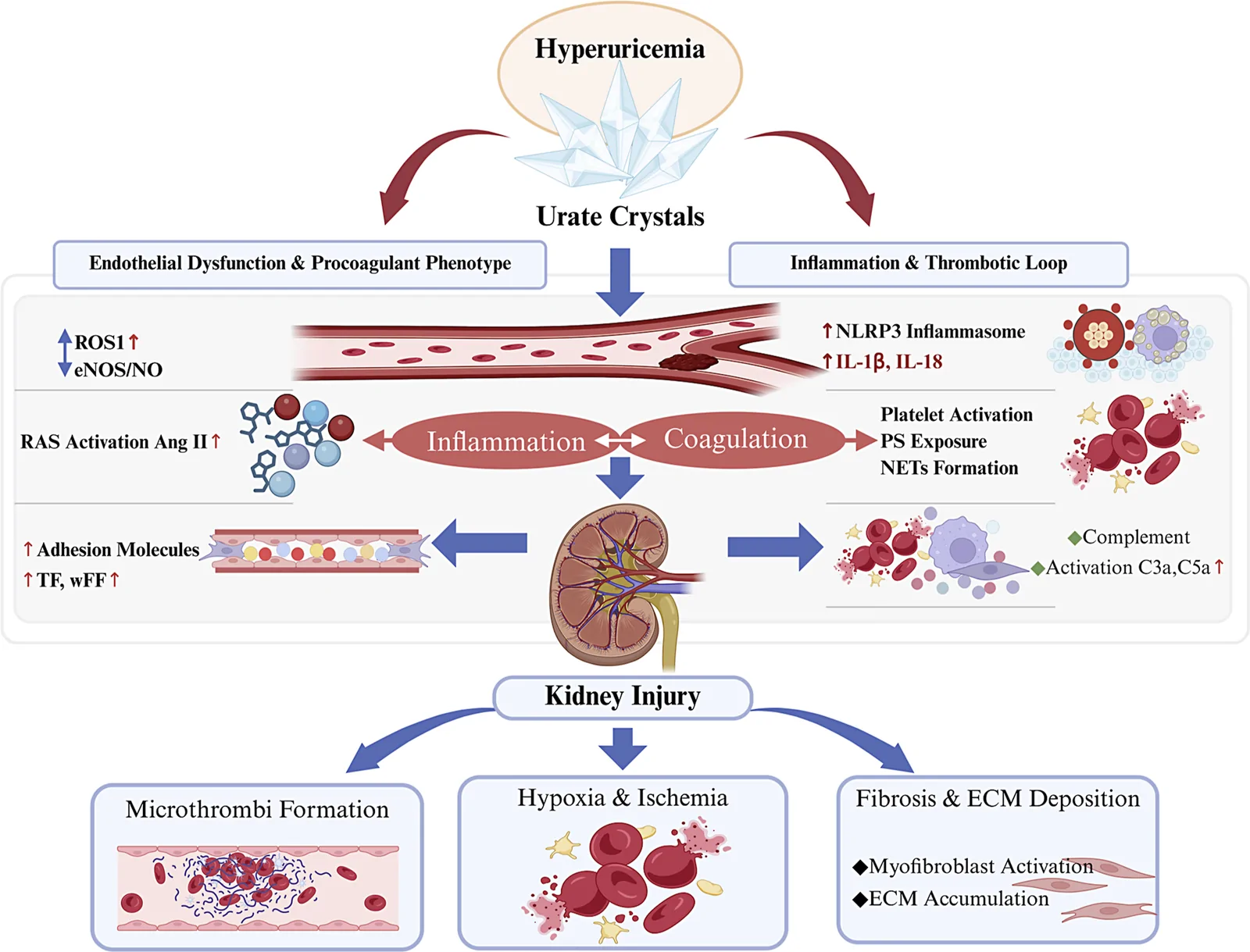
Pathogenic role of the thromboinflammation network in HN. This figure illustrates the central role of the thromboinflammation cycle in the progression of HN. Hyperuricemia initiates endothelial dysfunction, which promotes a pro-inflammatory and procoagulant state. The resulting activation of platelets, coagulation factors, and the NLRP3 inflammasome creates a self-amplifying cycle. This leads to intrarenal microthrombosis, ischemia, hypoxia, and ultimately progressive renal fibrosis.

### Central Hub: Endothelial Dysfunction and Procoagulant Phenotypic Transition

The vascular endothelium is a key barrier in maintaining blood fluid homeostasis. Elevated uric acid impairs endothelial function through multiple mechanisms, driving its transformation into a procoagulant and pro-inflammatory phenotype.

#### Nitric Oxide Inhibition and Oxidative Stress

Oxidative stress serves as a pivotal upstream driver of the thromboinflammatory cascade in HN. Soluble uric acid potently induces endothelial oxidative stress by directly stimulating oxygen species (ROS) overproduction [17]. A key consequence is endothelial nitric oxide synthase uncoupling, which shifts enzymatic function from vasoprotective nitric oxide generation to superoxide production, creating a vicious cycle that depletes bioactive nitric oxide (NO) and exacerbates oxidative injury [17]. This NO deficiency impairs endothelial vasodilation and weakens antithrombotic function. The ROS-rich milieu, amplified by monosodium urate (MSU) crystals, activates stress kinases (extracellular signal-regulated kinase 1/2, p38 mitogen-activated protein kinase) to upregulate inflammatory mediators [18, 19], ultimately promoting NLRP3 inflammasome activation and IL-1β release [18–20]. Beyond endothelium, oxidative stress enhances platelet activation and procoagulant phosphatidylserine exposure, drives eryptosis in red blood cells, and activates renal parenchymal cells to promote pro-fibrotic transformation. Clinically, hyperuricemia independently predicts endothelial dysfunction in CKD, while urate-lowering interventions improve endothelial function, highlighting this pathway’s therapeutic relevance [21].

#### Activation of the Renin-Angiotensin System

Uric acid can directly stimulate the local RAS in vascular endothelial and vascular smooth muscle cells, promoting the expression of angiotensin II, inducing microvascular damage and oxidative stress; it also directly upregulates the expression of procoagulant factors, establishing a vicious cycle. RAS inhibitors can partially block this process [22].

#### Activation of Pro-Inflammatory and Procoagulant Phenotypes

Damaged endothelial cells upregulate adhesion molecules and chemokines via pathways such as nuclear factor-kappa B (NF-κB), recruiting inflammatory cells. Concurrently, they upregulate tissue factor and release von Willebrand factor (vWF) and P-selectin while downregulating thrombomodulin, thereby initiating coagulation and impairing physiological anticoagulation [17]. Following endothelial damage, interactions with activated platelets exacerbate the process: endothelin-1 and platelet-activating factor promote vasoconstriction, whereas tissue factor, plasminogen activator inhibitor-1 (PAI-1), and coagulation factor V drive thrombosis [23].

### Bidirectional Vicious Cycle between Inflammation and Coagulation

Inflammation initiates and amplifies coagulation: systemic inflammation is a potent prothrombotic stimulus that upregulates procoagulants, inhibits anticoagulation and fibrinolysis, and enhances platelet reactivity [24]. The inflammatory state disrupts the coagulation system by promoting coagulation, inhibiting fibrinolysis, and weakening anticoagulant mechanisms, making it a major risk factor for venous thrombosis [25]. MSU crystals are the strongest known activators of the NLRP3 inflammasome, leading to a burst of cytokines such as IL-1β and IL-18. By activating immune cells such as neutrophils, they induce the release of ROS and neutrophil extracellular traps (NETs), thereby inducing tissue factor expression and powerfully initiating the coagulation system.

Coagulation reciprocally amplifies inflammation: activation of the coagulation pathway itself can exacerbate inflammation [26]. Activated coagulation factors, such as FXa, are potent inflammatory signaling molecules. By binding to PARs on the surface of endothelial cells and other cells, they activate mitogen-activated protein kinase and the NF-κB pathway. This subsequently triggers NLRP3 inflammasome assembly and activation [6], establishing a self-amplifying “inflammation → coagulation → re-inflammation” loop. Stimuli such as hypoxia can also upregulate NLRP3 via the hypoxia-inducible factor-1α (HIF-1α) pathway and drive thrombosis [27], potentially synergistically amplifying this cycle in the ischemic-hypoxic environment caused by intrarenal microthrombi.

### Involvement of Blood Cells and the Complement System

#### Platelet Activation and Hyperfunction

In patients with gout, particularly during the acute phase, plasma levels of platelet activation markers such as soluble glycoprotein VI and P-selectin are significantly elevated [28], suggesting the presence of chronic platelet hyperreactivity. Activated platelets not only participate directly in thrombosis, but the contents of the granules they release are potent pro-inflammatory and procoagulant substances that amplify the inflammation-coagulation cascade. Furthermore, high uric acid levels can promote vascular smooth muscle proliferation and upregulate platelet-derived growth factor [29], which may be associated with platelet hyperreactivity.

#### Active Procoagulant Role of Red Blood Cells

Studies have shown that in patients with hyperuricemia and in red blood cells cultured in vitro, elevated uric acid levels can lead to the exposure of PS on the red blood cell surface and the release of microparticles (MPs) [30, 31]. PS exposure and MP formation facilitate assembly of the prothrombinase complex, promoting thrombin generation and the formation of a dense fibrin network. Hyperuricemia may mediate PS inversion and MP shedding by increasing oxidative stress in erythrocytes and activating the calcium-dependent transmembrane protein 16F (TMEM16F) channel [32]. It is worth noting that PS exposure is not only a key step in the procoagulant activation of vascular cells but also a hallmark of erythrocyte programmed cell death (eryptosis). Red blood cells from patients with hyperuricemia exhibit a greater tendency for PS exposure [33], which provides insight into understanding how hyperuricemia exacerbates thrombotic risk by promoting erythrocyte apoptosis; this effect is particularly pronounced in the presence of comorbidities such as diabetes, renal insufficiency, and metabolic syndrome [34, 35]. Structural characteristics of thrombi, such as fiber diameter and density, influence their formation and clinical outcomes [36]. Fibrin thrombi characterized by fine fibrils and low porosity are typically denser and less permeable [37]. Yan et al. [38] found that uric acid-treated erythrocytes and the MPs they release are more prone to forming dense, highly branched fibrin thrombus networks, which exhibit reduced permeability and are more susceptible to fibrinolytic dissolution. These findings suggest that erythrocytes and their MPs are involved in hyperuricemia-associated thrombosis; uric acid activates the TMEM16F channel via oxidative stress, promoting a prothrombotic phenotype in erythrocytes [38]. Erythrocyte surface PS, circulating procoagulant MPs, and the TMEM16F channel may serve as novel biomarkers for assessing thrombotic risk in patients with hyperuricemia.

#### Cross-Activation of the Complement System and NTEs

The complement system critically amplifies thromboinflammation. MSU crystals activate complement, generating C3a and C5a, which recruit and prime neutrophils. Activated neutrophils release NETs, which serve as a scaffold for platelet adhesion and fibrin deposition, promoting the formation of “immunothrombi.” NET components also directly activate the intrinsic coagulation pathway and damage endothelium. This process creates a vicious cycle as coagulation proteases (e.g., thrombin, FXIIa) can in turn cleave and activate complement components. The sustained activation of this complement-coagulation-NET axis drives renal microvascular injury and the inflammatory milieu that fuels fibrosis in HN.

### Local Integrative Effects in the Kidney: Microthrombosis, Hypoxia, and Progressive Fibrosis

Due to its unique physiological structure – high blood flow, a dense glomerular capillary network, and the high metabolic demands of renal tubular epithelial cells – the kidney is particularly vulnerable to systemic prothrombotic or “coagulopathy-microthrombosis” states. This renders the renal microcirculation a primary site for pathological cascades associated with chronic inflammation (as seen in HN).

#### Induction of Intrarenal Microthrombosis and Hypoxia

The systemic pro-inflammatory and procoagulant environment in advanced gout converges within the renal microvasculature, leading to diffuse microthrombosis in the glomeruli and peritubular capillaries [39]. These microthrombi directly cause ischemia and hypoxia at the nephron level. The resulting hypoxic microenvironment stabilizes HIF-1α, which, as a key transcriptional regulator, amplifies the local inflammatory response and upregulates pro-fibrotic genes, creating a vicious cycle [40].

#### Activation of Fibrogenic Pathways and Progressive Fibrosis

Concurrently, the local release of ischemic and inflammatory signals, together with coagulation by-products (such as thrombin), constitutes a potent stimulus that activates resident fibroblasts and promotes the phenotypic conversion of peritubular cells and epithelial/endothelial cells into myofibroblasts capable of producing extracellular matrix (ECM). These activated cells excessively synthesize and deposit ECM components (primarily collagen). This pathological ECM accumulation manifests as glomerulosclerosis and tubulointerstitial fibrosis [41], representing the ultimate common pathway for progressive, irreversible loss of nephrons and functional decline in HN.

## Potential Mechanisms of AT in HN

The following sections detail the molecular actions of AT. It is important to clarify that while the basic biochemistry of AT and its protective effects in other models of kidney injury are well-established, its specific role and therapeutic mechanisms in HN remain hypothetical and have not yet been directly proven. The discussion in this chapter constructs a plausible mechanistic framework for AT in HN by integrating three key elements: the established pathophysiology of HN, which includes endothelial dysfunction, thromboinflammation, and microthrombosis; the well-characterized molecular and cellular functions of AT, such as its anticoagulant, anti-inflammatory, and endothelial-protective properties; and evidence of AT’s efficacy in other renal injury models that share similar pathological features, such as acute kidney injury (AKI) and ischemia-reperfusion injury (IRI). Mechanisms that are explicitly supported by studies in HN models will be highlighted accordingly.

### Molecular Structure and Anticoagulant Mechanism

AT is the most critical physiological inhibitor of coagulation factors (Fig. 2). It is a vitamin K-independent serine protease inhibitor synthesized by the liver. Its primary function is to inhibit the coagulation cascade, most potently by inactivating thrombin (FIIa) and FXa [42]. Structurally, AT resembles α1-antitrypsin but exhibits far superior activity against thrombin. Furthermore, it inhibits other serine proteases in the coagulation cascade, including factors XIIa, XIa, IXa, and Xa. The inhibitory activity of AT is markedly accelerated upon binding to heparin, a negatively charged glycosaminoglycan present on endothelial cell surfaces and mast cells. This binding induces a conformational change in AT, greatly accelerating the formation of stable, irreversible complexes with its target proteases, thereby amplifying its anticoagulant effect [43].

**Fig. 2.**
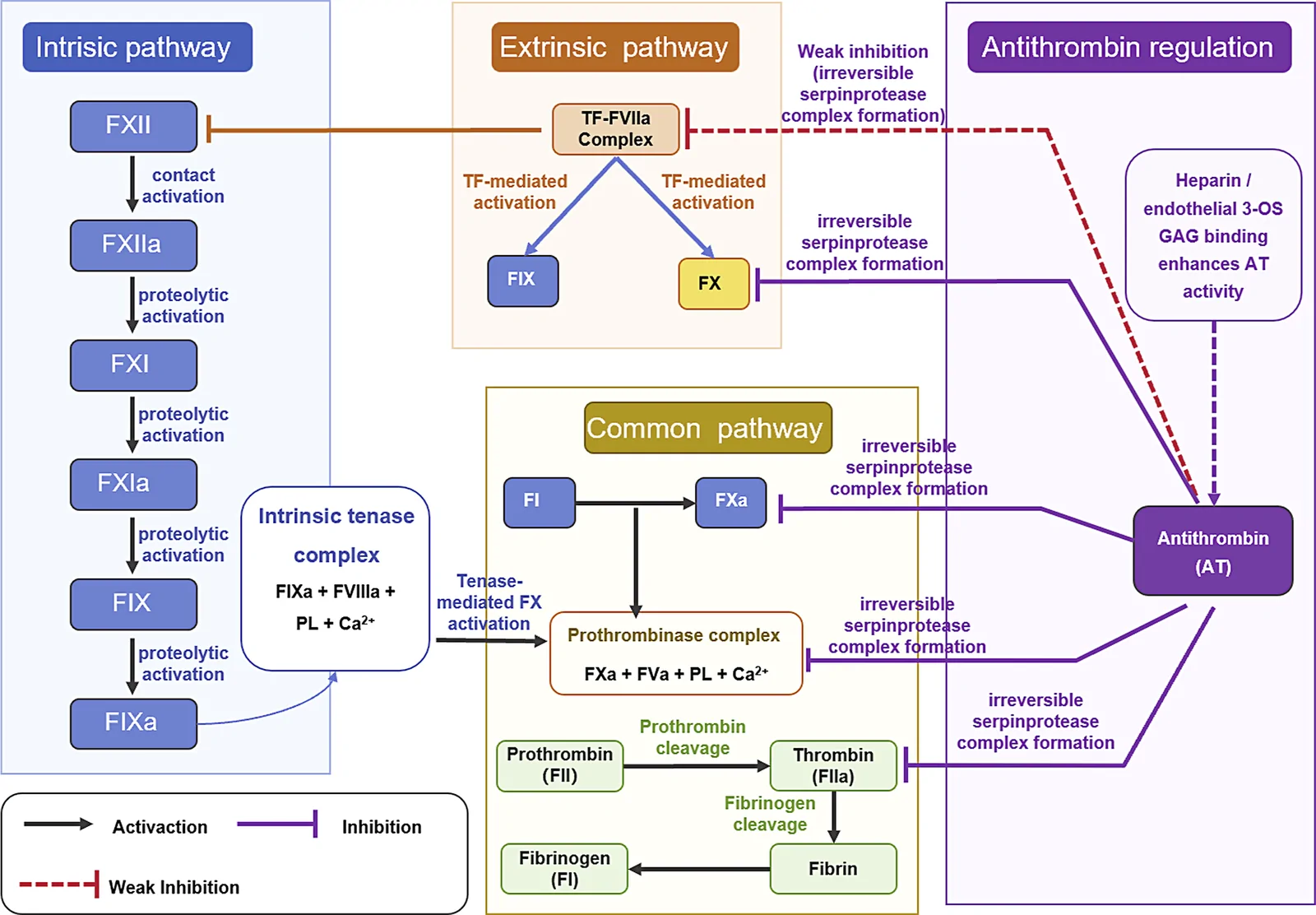
Mechanism of action and structure-function relationship of antithrombin (AT). AT is a serine protease inhibitor (serpin) and a key physiological anticoagulant. Its inhibitory activity against coagulation factors, most potently thrombin (FIIa) and factor Xa (FXa), is greatly accelerated upon binding to heparan sulfate proteoglycans on the endothelium or to therapeutic heparin. This conformational change enhances the formation of a stable, inactive enzyme-inhibitor complex, thereby suppressing coagulation.

The molecular function of AT depends critically on two distinct binding sites: one for the active site of target coagulation factors (notably thrombin) and another for heparin or cell-surface glycosaminoglycans containing specific modifications (e.g., 3-O-sulfated heparan sulfate) [10, 44, 45]. Although AT possesses intrinsic inhibitory activity, its binding to heparin induces a conformational change that dramatically enhances this activity [10]. AT can inactivate a broad spectrum of coagulation factors, including those of the intrinsic (contact) pathway (e.g., FXIa, FXIIa, kallikrein), with its highest affinity reserved for FXa and thrombin. Upon binding, it forms a stable covalent complex, irreversibly inactivating the enzyme. The physiological importance of AT is underscored in deficiency states; for instance, mutations in the SERPINC1 gene encoding AT can significantly reduce its inhibitory capacity against thrombin, increasing thrombotic risk [46–48].

### Anti-Inflammatory Activity and Immunomodulatory Effects: Insights from Other Disease Models

AT exhibits anti-inflammatory properties in the treatment of sepsis: as early as 1998, a prospective study found that long-term supplementation with AT could alleviate the systemic inflammatory response in patients with severe sepsis and reduce levels of inflammatory markers such as IL-6 and CRP [49]. In heparinized whole blood, AT downregulates the expression of IL-6 and IL-8 within monocytes [50] and significantly inhibits LPS-induced secretion of TNF-α by monocytes; when combined with beprastone, it exhibits a synergistic anti-inflammatory effect [51]. AT also downregulates the expression of pro-inflammatory integrins following the activation of neutrophils and monocytes [52]. A 2001 multicenter, randomized, double-blind, placebo-controlled trial was the first to evaluate the therapeutic efficacy of high-dose AT on a large scale in patients with high-risk severe sepsis [53]. The study indicated that, although there was no statistically significant difference in overall mortality, there was a significant trend toward reduced 28-day mortality in the AT treatment group within the subgroup of patients not receiving heparin. In 2006, Wiedermann et al. [12] conducted a further in-depth analysis of the data from this JAMA study. The study focused specifically on the patient subgroup at highest risk of death. The analysis revealed that, among these patients, high-dose AT treatment significantly improved 28-day survival rates compared with the placebo group. These studies collectively support the notion that AT treatment demonstrates a tendency to improve microcirculatory perfusion, mitigate organ damage, and reduce mortality.

Since the discovery that AT possesses anti-inflammatory effects independent of its anticoagulant action [54], experiments have confirmed two mechanisms: (1) AT induces the release of prostacyclin *I*^2^ from endothelial cells by binding to heparan sulfate proteoglycans (HS-PGs) (such as syndecan-4 [55]), thereby inhibiting platelet activation and leukocyte adhesion [56]; it may also interact with integrin receptors [57]. (2) AT directly binds to specific receptors, including low-density lipoprotein receptor-related protein 1 (LRP-1), CD13, and CD300f, reducing the expression of pro-inflammatory factors such as IL-6, TNF-α, and tissue factor by inhibiting pathways like NF-κB [11]. The anti-inflammatory activity of AT is mediated by its D-helical domain (which also serves as the heparin-binding site) [58]. Higher AT levels are associated with lower histone H3 levels, which may mitigate immune-mediated tissue damage [59]. Histone H3 possesses cytotoxic properties and can be released through pathways such as NETosis [60], the latter of which can be induced by activated platelets [61]; however, the direct effects of thrombin or AT on neutrophil activation and NETosis have not yet been clearly investigated.

### Correction of Endothelial Dysfunction

As the body’s most important natural anticoagulant, the therapeutic value of AT extends beyond neutralizing coagulation factors such as thrombin and FXa. Its multifaceted endothelial-protective and anti-inflammatory properties position it as a potential candidate for reversing the procoagulant phenotype. Under physiological conditions, AT significantly enhances the activity of HS-PGs by binding to their surface on endothelial cells, making it crucial for maintaining the anti-thrombotic capacity of the vascular wall. In chronic inflammatory states, including hyperuricemia, endothelial damage leads to impaired HS-PG expression and function, resulting in relative insufficiency of local AT activity. Supplementation with exogenous AT, particularly high-affinity recombinant human AT, has been shown to exert multifunctional effects beyond those of traditional anticoagulants: (1) Direct endothelial protection: AT inhibits thrombin-induced endothelial cell apoptosis and mitigates ROS-mediated oxidative stress injury, thereby stabilizing endothelial barrier function [62]. (2) Promotion of prostacyclin release: early studies found that AT, through its interaction with endothelial cells, induces the synthesis of prostacyclin, exerting potent vasodilatory and antiplatelet effects. Moreover, AT-induced prostacyclin-I-2 (PGI_2_) production is distinctly different from thrombin-induced PGI_2_ production [45]. Consequently, viewing AT as a multifunctional drug aimed at “reshaping the anticoagulant endothelial phenotype” offers a novel research direction for exploring interventions against kidney injury driven by endothelial dysfunction.

### Potential for Intervening at Multicellular Targets

As previously mentioned, in HN, various blood cells and NETs constitute a complex, self-amplifying “inflammation-coagulation” network. A core feature of this network is the exposure of procoagulant substances on the cell membrane surface (such as platelet P-selectin and erythrocyte PS) and the release of pro-inflammatory/procoagulant granules. In addressing this pathological network, AT therapy demonstrates a unique cell-targeting effect that transcends the mere inhibition of liquid-phase coagulation factors. The rationale is as follows.

#### Direct Neutralization of Cell-Derived Procoagulant Activity

Kambas et al. [63] established a key role for AT in inhibiting “cell-dependent” coagulation amplification through the development of an in vitro functional model. First, the researchers confirmed that, under specific inflammatory pathological conditions, extracellular traps and granules released by activated neutrophils, whose surfaces are rich in tissue factor and expose PS, can act as highly efficient procoagulant platforms, significantly accelerating the burst-like generation of thrombin. Subsequently, in intervention experiments with exogenous AT, thrombin generation assays showed that AT significantly delayed the onset of thrombin generation and reduced its peak levels. This result demonstrates that AT, through its classic dual anticoagulant mechanism – highly efficiently neutralizing preformed thrombin and inhibiting the upstream key protease FXa – is able to directly block the cascade reactions occurring on procoagulant cell surfaces, thereby suppressing pathological coagulation bursts driven by activated blood cells and their products at the source.

#### Inhibition of NET-Mediated Coagulation

The formation of NET is thought to contribute to the formation and propagation of thrombosis in arterial, venous, and cancer-associated thrombosis. A review published in Arteriosclerosis, Thrombosis, and Vascular Biology in 2019 concluded [64] that the activation products of the coagulation cascade (such as thrombin) are themselves potent inducers of NET release. Therefore, conventional anticoagulants represented by heparin, low-molecular-weight heparin, and direct oral anticoagulants can attenuate key upstream signals for NET release by inhibiting thrombin or FXa, thereby reducing NET formation. At the same time, the review also explores more specific potential targets, such as the development of inhibitors against peptidyl-arginine deiminase 4 or neutrophil elastase, to achieve precise intervention further upstream of NET formation for the prevention and treatment of NET-dependent thrombosis.

### Potential Significance of at Antagonizing Thrombin Signaling: Blocking Multiple Fibrogenic Signals

The formation of intrarenal microthrombi is not only a direct cause of local ischemia; the mediators released by their persistent presence, such as thrombin, also serve as a continuous source of stimulation driving inflammation and fibrosis. AT represents a candidate agent that may exert both anti-inflammatory and cell-protective effects.

#### Intervening at the Source to Prevent Microthrombus Formation and Improve Renal Hypoxia

By potently inhibiting thrombin and FXa, AT can block the abnormal deposition of fibrin and the expansion of microthrombi in the glomeruli and peritubular capillaries at an upstream level. In animal models of IRI and sepsis-associated AKI, exogenous AT supplementation has been shown to reduce intrarenal fibrin deposition and improve renal cortical blood flow and oxygenation [65]. This provides a direct theoretical basis for its potential to alleviate renal unit ischemia and hypoxia caused by microthrombi in HN.

#### Direct Antagonism of the “Multifaceted” Fibrogenic Effects of Thrombin

Thrombin released during local microthrombus formation can directly activate renal interstitial fibroblasts and renal tubular epithelial cells via PARs (such as PAR-1), promoting their transformation into myofibroblasts [66]. By neutralizing thrombin, AT may directly antagonize the pro-fibrotic signals mediated by these receptors, thereby offering a potential mechanistic avenue to intervene in the initiation of fibrosis [67]. These effects help to break the “hypoxia-inflammation-re-injury” cycle, creating a microenvironment in the kidney that is unfavorable to the progression of fibrosis. A graphical summary of the proposed mechanisms of action of AT in HN, integrating known pathophysiology and hypothesized interventions, is depicted in Figure 3. It should be noted that the above discussion on AT’s potential to block fibrogenic signals represents a hypothesis constructed by combining the known pro-fibrotic role of thrombin in kidney disease and the established thrombin-neutralizing function of AT. Direct evidence for this mechanism in HN models is currently lacking.

**Fig. 3.**
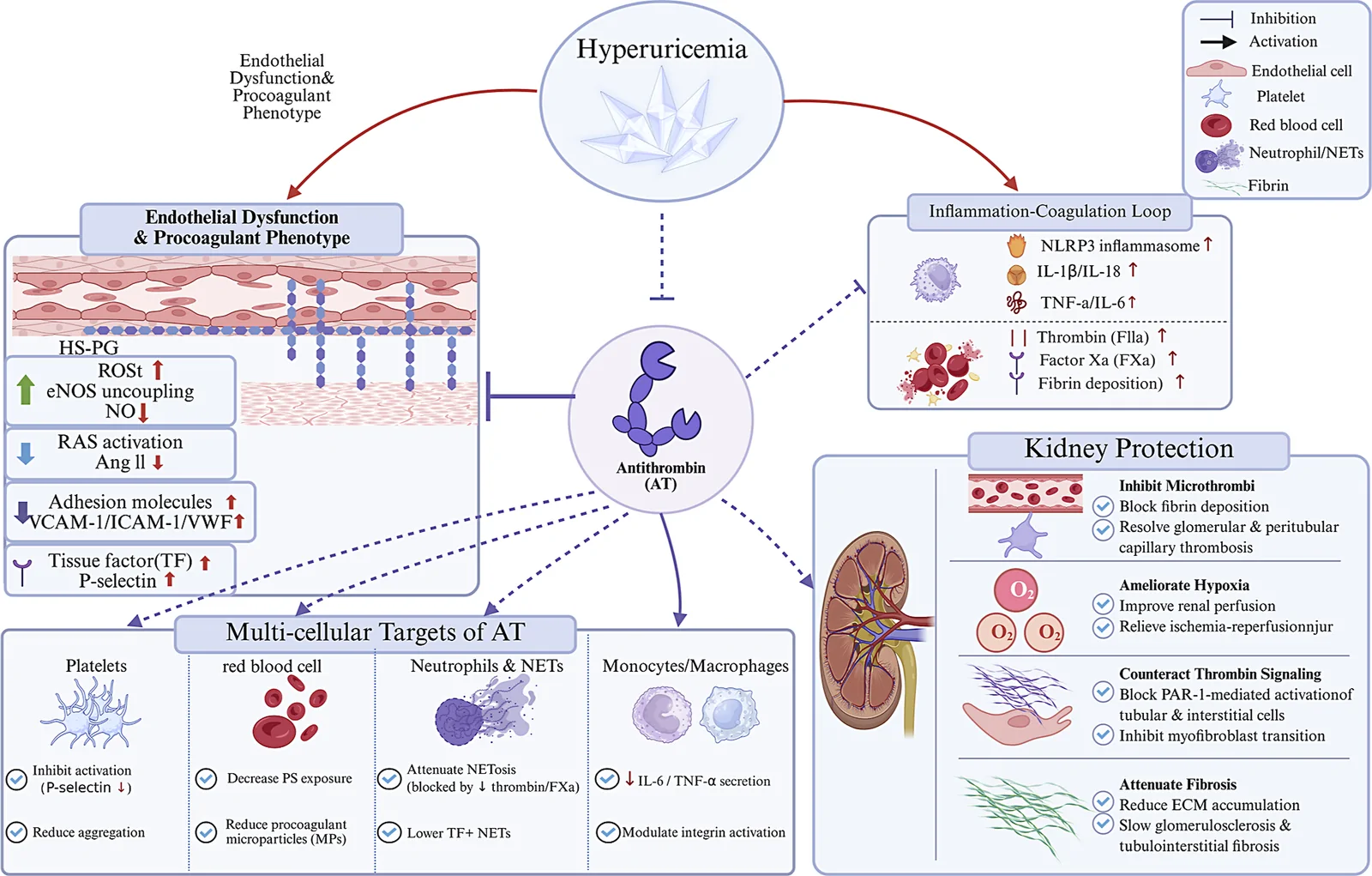
Proposed renoprotective mechanisms of antithrombin (AT) in hyperuricemic nephropathy (HN). A graphical summary of the hypothesized mechanisms of AT in HN. The figure integrates (i) the established pathophysiology of HN (grey boxes) and (ii) the proposed interventions of AT (blue boxes/arrows). Solid blue arrows represent mechanisms of AT action that are well-documented in other kidney disease models and in vitro studies (e.g., inhibition of thrombin/FXa, induction of prostacyclin). Dashed blue arrows represent mechanisms that are plausible extrapolations to the specific context of HN (e.g., NLRP3 inflammasome inhibition, NETosis blockade) and require direct experimental validation in HN models.

## Evidence for Anticoagulant Therapy in Kidney Disease

### Renal Protection Conferred by Direct Factor Xa Inhibitors

Direct FXa inhibitors (such as rivaroxaban, edoxaban, and apixaban) have demonstrated clear anti-inflammatory and anti-fibrotic renal protective effects in CKD models, with mechanisms that extend beyond simple anticoagulation. A summary of the protective effects of direct FXa inhibitors and other anticoagulants, including AT, across a spectrum of kidney injury models and clinical contexts is provided in Table 1. Evidence from basic research: in animal models of CKD, such as diabetic nephropathy, glomerulonephritis, and unilateral ureteral obstruction in mice, FXa inhibitors significantly reduce proteinuria and alleviate renal tissue inflammation, ECM deposition, and fibrin microthrombus formation [7]. Mechanistic studies indicate that rivaroxaban inhibits NLRP3 inflammasome activation by blocking the FXa-PARs-MAPK/NF-κB pathway, thereby reducing vascular inflammation and dysfunction [6]. Knockout of the NLRP3 gene similarly alleviates FXa-induced endothelial dysfunction. In a 5/6 nephrectomy CKD model, edoxaban treatment inhibits FXa and blocks its downstream ERK/NF-κB signaling, reducing proteinuria and fibrin deposition and alleviating tubulointerstitial fibrosis [68]. Clinical research findings: in patients with diabetic nephropathy, treatment with oral sulodate (a glycosaminoglycan with anti-Xa activity) and low-molecular-weight heparin both significantly reduced proteinuria, independent of changes in blood pressure or the renin-angiotensin-aldosterone system (RAAS) [8, 69]. Real-world big data analyses provide even more compelling evidence. A study involving over 70,000 patients with non-valvular atrial fibrillation showed that, compared with warfarin, rivaroxaban treatment reduced the risk of AKI by 19% and reduced the risk of progression to end-stage renal disease or the need for dialysis by 18% [70]. A prospective observational study further found that, in patients with stage 3b–4 CKD and atrial fibrillation, 24 months of rivaroxaban treatment, compared with warfarin, significantly improved renal function, reduced levels of inflammatory markers, and was associated with stabilization or reversal of cardiac valve calcification [9]. Taken together, this evidence suggests that FXa inhibitors may exert a renoprotective effect in CKD (including nephropathy with underlying hyperuricemia) characterized by a hypercoagulable and inflammatory background through their multifactorial “anticoagulant-anti-inflammatory-anti-fibrotic” mechanisms.

**Table 1.** Summary of renoprotective evidence and core mechanisms of anticoagulants in kidney disease models and clinical studies

Category	Direct factor Xa inhibitors		AT		
Drug/ingredient	Rivaroxaban, edoxaban, apixaban, etc.	​	AT	​	​
Nephropathy model/clinical study	Basic research (diabetic nephropathy, glomerulonephritis, UUO, 5/6 nephrectomy model)	Clinical studies (patients with diabetic nephropathy and atrial fibrillation with CKD)	AKI models (ischemia-reperfusion, rhabdomyolysis, contrast-induced nephropathy, sepsis, etc.)	CKD model (AKI-to-CKD transition model) factors	Clinical observations (nephrotic syndrome, critically ill patients)
Key evidence/findings	Significantly reduces proteinuria, renal tissue inflammation, fibrosis, and microthrombosis	1. Reduces proteinuria (surotide/low-molecular-weight heparin) 2. Reduces the risk of AKI and delays progression to ESRD (rivaroxaban vs. warfarin) 3. Improves renal function, reduces inflammatory markers, and stabilizes cardiac valve calcification (CKD stages 3b–4)	1. Exogenous supplementation of AT significantly reduces kidney injury and lowers creatinine and blood urea nitrogen levels 2. Improves renal blood flow, reduces leukocyte infiltration, and alleviates oxidative stress and apoptosis 3. Protects the endothelial glycocalyx	Exogenous AT supplementation improves renal function, reduces renal interstitial fibrosis, and inhibits M1 macrophage infiltration and pro-inflammatory response	1. Low plasma AT activity is an independent risk factor for AKI and poor prognosis 2. Urinary AT levels are associated with declining renal function (e.g., diabetic nephropathy) 3. AT replacement therapy is effective for patients with nephrotic syndrome and thrombosis who have AT deficiency
	Inhibits the FXa-PARs-MAPK/NF-κB pathway and NLRP3 inflammasome activation				
Core protective mechanism	Effects extending beyond anticoagulation: Anti-inflammatory: Inhibits inflammatory pathways such as NLRP3 Anti-fibrotic: reduces ECM deposition Endothelial protection: improves vascular dysfunction	​	Targets the core mechanisms of “thrombosis inflammation”: Anticoagulant: inhibits the coagulation cascade Anti-inflammatory: induces prostacyclin *I*^2^ release and inhibits leukocyte activation Cell protection: provides antioxidant and anti-apoptotic effects	​	​

The evidence summarized herein is derived from studies in various kidney disease models (e.g., diabetic nephropathy, IRI, sepsis AKI) and clinical conditions, not specifically from HN models. The therapeutic potential of these anticoagulants in HN remains hypothetical, based on extrapolation from their efficacy in pathologically analogous conditions.

UUO, unilateral ureteral obstruction; ESRD, end-stage renal disease.

### Renal Protective Effects of AT

#### Protective Effects in AKI Models

AT has been shown to exert a protective effect in various etiology-induced AKI models [71]. Studies have found that following renal IRI, the coagulation system is rapidly activated, with AT levels declining within 2 h, reaching a minimum at 12 h, and subsequently beginning to rise again [72]. Wang and colleagues [73] found that patients with lower AT activity had a higher risk of developing AKI following cardiac surgery. Furthermore, in a rat model with heterozygous SerpinC1 deficiency, it was found that AT deficiency exacerbates renal IRI. The increase in the prostacyclin metabolite F1α was significantly attenuated, and this attenuation preceded the worsening of tubular damage and oxidative stress, suggesting that the protective effect of AT may be mediated by prostacyclin *I*^2^ released from endothelial cells [73]. Current research indicates that exogenous AT supplementation can significantly alleviate renal injury through mechanisms including improved renal blood flow and reduced vascular permeability; inhibition of leukocyte (particularly neutrophil) activation and infiltration via the induction of PGI_2_ release; and a reduction in lipid peroxidation products [65, 73]. In various models, including rhabdomyolysis, contrast-induced nephropathy, AKI associated with severe acute pancreatitis, and sepsis-associated AKI, AT treatment has demonstrated similar protective effects – reducing serum creatinine and blood urea nitrogen, alleviating renal tissue inflammation, oxidative stress, and cell apoptosis, and promoting renal tubular epithelial cell regeneration and glomerular endothelial protection [65, 74–77]. Further studies have found that AT can prevent sepsis-associated respiratory and renal failure; the mechanism may lie in inhibiting the initiation of coagulation, reducing fibrin deposition, and controlling inflammation, thereby alleviating lung and kidney injury [78]. This evidence raises the possibility that the protective effects of AT may be broad spectrum, potentially targeting the common pathological pathway of “thromboinflammation” in relevant disease contexts.

#### Evidence in the Progression of CKD and Nephrotic Syndrome

In a rat model of AKI-to-CKD transition established via “renal IRI combined with nephrectomy,” exogenous AT supplementation significantly improved renal function and reduced renal tissue damage, as well as mitigating renal interstitial fibrosis. The mechanism is associated with the inhibition of M1 macrophage infiltration and the downregulation of pro-inflammatory factors such as TNF-α, iNOS, and IL-1β [65], demonstrating AT’s capacity to intervene in the chronic fibrosis process. Pahl et al. [79] found in vitro that thrombin stimulates DNA synthesis and cell growth in cultured human glomerular mesangial cells. Both standard heparin and AT, when used alone or in combination, were able to inhibit the effects of thrombin on DNA synthesis and mesangial cell proliferation, suggesting a protective role in glomerulonephritis. Patients with nephrotic syndrome, due to massive proteinuria leading to AT loss, are in a hypercoagulable state and are prone to thrombosis [80]. A case report describes a patient with nephrotic syndrome who also had arterial thrombosis and AT deficiency; following thrombectomy, the patient received 1,000 units of AT as anticoagulant therapy, achieving good results with no recurrence [81].

#### As a Key Biomarker for Risk Prediction and Stratification

Numerous observational clinical studies have established the value of AT levels in predicting the occurrence and prognosis of AKI. In various clinical settings, including sepsis, post-liver transplantation, traumatic brain injury, and pre-eclampsia, low plasma AT levels are an independent risk factor for AKI [82–85]. A recent retrospective study found that in patients with sepsis, AT activity <55 % (general patient population) or <64% (hypertension subgroup) was an independent risk factor for significantly increased 28-day mortality, disseminated intravascular coagulation, and AKI incidence. This suggests that AT activity may serve as a key biomarker for prognostic stratification in sepsis, providing clear cut-off values for identifying high-risk patients [86]. In patients with acute glomerulonephritis and nephrotic syndrome, reduced plasma AT activity and elevated urinary AT levels have been observed [87]. In patients with diabetic nephropathy, urinary AT levels are associated with declining renal function [88]. This provides a clue for exploring AT as a biomarker of “thrombotic-inflammatory” activity in HN.

### Emerging Genetic and Multi-Omic Evidence in Humans: Preliminary Insights from SERPINC1 Mendelian Randomization

Direct, human-level genetic support for this rationale is provided by our latest pre-publication study (currently under peer review). Through integrative multi-omics mendelian randomization, we have uncovered preliminary genetic insights connecting SERPINC1 (encoding AT) to the hyperuricemia-kidney axis, offering exploratory evidence bridging preclinical mechanisms to human pathophysiology. Key findings relevant to HN – which remain to be validated in independent cohorts – include a genetically suggested bidirectional relationship between hyperuricemia and kidney dysfunction, supporting the self-reinforcing pathogenic cycle central to HN. The SERPINC1 gene appeared to act as a disease-phase responder rather than a primary genetic mediator, showing dynamic expression patterns with compensatory upregulation during acute flares and potential downregulation in chronic hyperuricemia. Multi-omic analyses tentatively positioned SERPINC1/AT as a network hub, linking metabolic disturbances to renal stress pathways. Notably, these observations are preliminary and should be interpreted with caution until corroborated by external datasets and prospective clinical studies.

Collectively, these preliminary human genetic findings provide tentative support for the therapeutic hypothesis for AT in HN: they suggest that AT may function as a molecular responder within the thromboinflammatory network, and its consumption-like decrease in chronic states warrants further investigation into AT activity as a potential dynamic biomarker. Consequently, these data raise the possibility that AT supplementation could be considered beneficial in a carefully selected subset of patients with functional AT deficiency or high thromboinflammatory burden, pending confirmation in future HN-specific studies.

## Implications for Anticoagulant Therapy in HN

Given the protective role of AT in other renal disease models and the potential correlation between its mechanism of action and certain pathological features of HN (such as endothelial damage, inflammation, and hypercoagulability), it is reasonable to consider investigating AT supplementation therapy in HN in the future. The chronic “thrombotic-inflammatory” state in HN can lead to “acquired relative AT deficiency” due to continuous consumption and loss via proteinuria. This state poses a dual threat. First, it weakens the anticoagulant barrier, which accelerates the formation of intrarenal microthrombi. Second, it diminishes the inhibition of the pro-inflammatory activity of coagulation factors, such as FXa. Consequently, AT deficiency might disinhibit the “inflammation-coagulation” vicious cycle, thereby potentially contributing to renal fibrosis.

It is worth noting that in the field of cardiac surgery, although observational studies suggest that low AT activity post-surgery is associated with adverse outcomes, randomized controlled trials indicate that blind supplementation with exogenous AT fails to improve prognosis and may even increase the risk of AKI [89]. This suggests that postoperative low AT activity may serve more as a marker of disease severity rather than a correctable etiological factor. Consequently, when investigating the role of AT in HN, the primary research objective should be to determine whether low AT activity constitutes a target for intervention or merely a biomarker reflecting the “thrombotic-inflammatory” burden; furthermore, the feasibility of targeted replacement therapy should only be explored in subgroups where severe AT deficiency and a hypercoagulable state have been confirmed via biomarkers. However, there is currently no direct evidence to suggest that AT has a definitive therapeutic effect in models of HN. Consequently, the primary research step should be to validate the efficacy of AT treatment in animal models and to investigate its specific mechanisms of action in depth. Furthermore, it is necessary to clarify whether the low AT activity observed in patients represents a potential therapeutic target or merely a biomarker reflecting disease severity. As summarized in Table 1, direct FXa inhibitors and AT demonstrate renoprotective effects across a spectrum of kidney injury models and in clinical settings, extending beyond their conventional anticoagulant roles.

## Summary, Clinical Translation, and Future Research Directions

This review establishes a “thromboinflammation” circuit as the core driver of HN, linking hyperuricemia-induced endothelial dysfunction, coagulation activation, and innate immunity to progressive renal fibrosis. This framework repositions the coagulation system – particularly the FXa-thrombin axis – as an active disease modifier. Evidence from analogous renal and thromboinflammatory conditions suggests that targeting this axis (e.g., with AT or direct FXa inhibitors) may confer renoprotection beyond anticoagulation [8, 9, 70]. This provides a plausible rationale for exploring the potential of these agents as candidate adjunctive, disease-modifying therapies in HN, subject to rigorous validation.

### Clinical-Translational Potential and Challenges

While the mechanistic rationale is strong, translating this into clinical practice for HN remains a hypothesis built primarily on extrapolation from other disease models. Current standard management strategies, such as urate-lowering therapy and RAAS blockade, often fail to completely halt the progression of renal fibrosis, highlighting the pressing need for novel therapeutic approaches. However, several specific hurdles must be overcome. First, there is a lack of HN-specific evidence: the efficacy and safety of anticoagulants, particularly AT, have yet to be validated in preclinical HN animal models. Second, risk-benefit management requires extreme caution due to the inherent bleeding risk in patients with CKD. Future interventions will likely depend on precise patient stratification using biomarkers of intrarenal thromboinflammation – such as phosphatidylserine-exposing erythrocytes or urinary fibrin fragments – to identify those most likely to benefit. Finally, prospective clinical validation is now an urgent priority to translate mechanistic insights into clinical utility. First, well-designed longitudinal cohort studies in HN or high-risk gout populations are required to definitively validate AT activity as a dynamic, noninvasive biomarker for risk stratification and disease monitoring. Following robust target validation in disease-specific preclinical models, proof-of-concept interventional trials should next be initiated in biomarker-stratified high-risk subgroups. Such trials should specifically enroll patients with confirmed functional AT deficiency or elevated thromboinflammatory burden to assess both safety and efficacy. Critically, these studies must prioritize hard renal endpoints, including the rate of eGFR decline and progression to end-stage renal disease, rather than relying solely on surrogate markers. Importantly, lessons from other fields – for example, the failure of AT supplementation in cardiac surgery [89] – remind us that compelling mechanistic rationale does not guarantee clinical success.

### Integrated Path Forward

Therefore, future research should follow a prioritized, sequential agenda to bridge the gap from mechanism to therapy, beginning with target validation: rigorously evaluating candidate agents (e.g., AT, FXa inhibitors, NLRP3/IL-1β inhibitors) in established HN animal models to assess disease-modifying effects and further elucidate HN-specific mechanisms. Concurrently, biomarker development is essential to identify and validate noninvasive biomarkers that enable precision patient selection for clinical studies. Following this, stratified clinical translation should initiate proof-of-concept trials in biomarker-stratified, high-risk HN subgroups, such as those with confirmed hypercoagulability or AT deficiency. While preliminary multi-omic analyses, including our own pre-publication mendelian randomization data, point to SERPINC1/AT as a responsive hub in this network, definitive validation in HN cohorts is lacking. Notably, HN shares the core “thromboinflammation” pathophysiology with atherosclerotic cardiovascular disease [90–92]. Consequently, therapeutic strategies targeting this network in HN – such as AT supplementation – may offer concurrent cardiorenal protection. This concept of pleiotropic benefit is strongly supported by the proven cardiovascular outcomes of colchicine, an established inhibitor of the same inflammatory pathway, providing a promising paradigm for drug repurposing in this context [93].

In summary, targeting the thromboinflammation network represents a promising potential frontier for HN. Translating this potential into clinical reality requires a concerted effort: from target validation in disease-specific models to the development of precision biomarkers and finally to stratified clinical trials that can definitively determine if quenching this pathological fire preserves kidney function.

## Acknowledgment

The authors would like to thank Yifan Wang of University of Wisconsin Madison for language improvement.

## Conflict of Interest Statement

No other specific funding was received from any funding body in the public, commercial, or not-for-profit sectors, and no conflicts of interest are declared.

## Funding Sources

This work was supported by the Shanghai General Hospital Discipline Construction Project (Grant No. 0206012418). The funding source provided financial support for the publication of this review, including the article processing charge, but had no role in the conception of the review, literature search, analysis, manuscript writing, or the decision to submit the manuscript for publication.

## Author Contributions

Conceptualization: L.J. and F.W.; methodology and visualization: L.J.; investigation and writing – original draft preparation: L.J. and X.-M. Z.; writing – review and editing, supervision, project administration, and funding acquisition: F.W. All authors have read and agreed to the published version of the manuscript.
